# Association of Serum 25-Hydroxyl Vitamin D Deficiency and Age-Related Cataract: A Case-Control Study

**DOI:** 10.1155/2019/9312929

**Published:** 2019-04-15

**Authors:** Marwa Mahmoud Abdellah, Engy Mohamed Mostafa, Eman Hassan Salama, Eman Roshdy Mohamed

**Affiliations:** ^1^Ophthalmology Department, Sohag University, Sohag, Egypt; ^2^Clinical Pathology Department, Sohag University, Sohag, Egypt; ^3^Public Health Department, Sohag University, Sohag, Egypt

## Abstract

**Purpose:**

To study the relation between the serum 25-hydroxyl vitamin D (OH D) level and the occurrence of age-related cataract in a case-control study.

**Patients and Methods:**

325 cataract patients and 385 control individuals of both sexes were examined for the 25-OH D level using the chemiluminescent microparticle immunoassay (CMIA) technology.

**Results:**

Mean 25-OH D level in cataract patients was 7.6 ± 5.5 ± 11.2 ng/mL and median was 5.6 (2.6–31.9), while in the control group, mean 25-OH D level was 18.5 ± 9.6 ng/mL and median was 17.8 (3.4–37.8) (*p* value < 0.001). There was a statistically significant difference among the different types of cataracts with the lowest level in nuclear cataract.

**Conclusion:**

25-OH D levels in all enrolled individuals were below the reference levels with a severe deficiency in cataract patients. These results might highlight the role of deficiency of 25-OH D in age-related cataract patients.

## 1. Introduction

Visual impairment constitutes a global challenge, especially for developing countries [1]. Visual impairment is one of the strongest risk factors for functional status decline in a community, rendering those individuals at higher risk of incapacitation and social isolation [2, 3].

Age-related cataract is the leading cause of blindness worldwide. The rising number of cataract patients outweighing the cataract surgical rate would lead to increased patients with visual impairment. In a cross-sectional study in four villages in Upper Egypt which were randomly selected, the prevalence of cataract was 22.9% with a higher prevalence in women (26.5%) than men (17.2%) [4].

Cataract is a multifactorial disease with age being identified as a major nonmodifiable risk factor, as well as brown iris color [5]. Smoking [6, 7], alcohol use [8], obesity [9], malnutrition, and phytochemicals [10] are modifiable risk factors that increase inflammation and oxidative stress. Vitamin D has been suggested to have anti-inflammatory properties [11], which might have a protective role against cataract formation [12]. Investigating the association between 25-OH D and its cataractogenesis effect might also be due to the fact that vitamin D suppresses oxidative stress [13] which can cause cataract.

Identifying a modifiable risk factor would help decrease or delay cataract in developing countries which make it a valuable and cost-effective step. In our study, we aim to detect a relationship between serum vitamin D status and the presence of cataract in ≥50-year-old patients.

## 2. Materials and Methods

The current study was conducted in the Ophthalmology Department of Sohag University Hospital, Sohag, Egypt, from April 2017 to December 2017 of 1222 cataract patients scheduled for cataract surgery in our department from April 2017 to December 2017; only patients meeting our inclusion criteria were included. Inclusion criteria are as follows: patients with age of 50 years or older, senile cortical, and nuclear and subcapsular cataract. Exclusion criteria are as follows: complicated cataract either to ocular or systemic cause, glaucoma, prior ocular medication or surgery, steroid use, use of calcium supplements or osteoporosis medications, prior trauma, autoimmune or skin cancer disorders, diabetes, cancer diagnosis, or cardiopulmonary disease. The control group was enrolled from the outpatient clinic following the same criteria with no cataract detected.

Lens opacity classification system (LOCS) was used for grading of both nuclear and cortical cataracts. Slit-lamp retroillumination was used to classify the type and grade of cataract [14]. A sample of 3 ml of peripheral blood was collected in a sterile plain vacutainer from 354 patients and 423 healthy controls for vitamin D assay; we excluded 29 cases from the case group (21 blood sample collections were not sufficient to complete the test, and 8 samples showed hemolysis), and 38 were excluded from control group (24 small samples and 14 hemolysis samples). In total, the case group included 325 patients and the control group included 385 participants. All serum samples were appropriately processed as serum samples and were obtained following centrifugation of whole blood after complete clot formation has taken place at 3000 rpm for 5 minutes and stored freezing at −80 degree for 2 months.

25-OH D was performed on an ARCHITECT i2000SR system using ARCHITECT 25-OH D kits supplied by Abbott. A chemiluminescent microparticle immunoassay (CMIA) technology was used to estimate the 25-OH vitamin D level.

Sample and pretreatment reagent are combined. An aliquot of the pretreated sample is combined with assay diluents and paramagnetic anti-vitamin D-coated microparticles to create a reaction mixture. Vitamin D present in the sample binds to anti-vitamin D-coated microparticles. After incubation, a biotinylated vitamin D anti-biotin acridinum-labelled conjugate complex is added to the reaction mixture and binds to unoccupied binding sites of the anti-vitamin D-coated microparticles. Washing followed by adding pretrigger and trigger solutions the reaction mixture, the resulting chemiluminescent reaction is measured as relative light units (RLUs). An indirect relationship exists between the amount of vitamin D in the sample and the RLUS detected by the ARCHITECT i2000SR system optics.

The capability of the ARCHITECT i2000SR system to measure ARCHITECT 25-OH D is wide, ranging from 8.0 ng/mL to 160 ng/ml. Samples having 25-OH D concentration greater than this value were diluted and then reanalyzed.

Reference ranges: the overall distribution of 25-OH D levels in both groups was stratified by 3 breakpoints <30 ng/mL, <20 ng/mL, and <10 ng/mL correlating with vitamin D insufficiency, deficiency, and severe deficiency, respectively [15].

Informed consent was obtained from patients and participants in both the cataract and control groups after explaining the aim of the study. The tenants of Helsinki were adhered, and the approval of the ethical committee of our institute was obtained.

## 3. Statistical Analysis

Data were analyzed using SPSS computer program version 22.0. Quantitative data were expressed as means ± standard deviation, median, and range. Qualitative data were expressed as number and percentage. The data were tested for normality using the Shapiro–Wilk test which was significant, indicating the data were not normally distributed which required nonparametric tests. The nonparametric Mann–Whitney test was used for comparing two quantitative variables; however, the Kruskal–Wallis test was used for comparison between more than two quantitative variables. The chi-squared test was used for comparison between qualitative variables, and Spearman's correlation was used for testing of correlation between different quantitative variables. The significance was determined by a level of 5% in all statistical used.

## 4. Results

In the cataract group (group 1), 325 patients were included, while the control group (group 2) had 385 patients. Patients with all morphological types of cataracts were enrolled: 105 (32.3%) patients with cortical cataract, 125 (38.5%) patients with nuclear cataract, and 95 (29.2%) with posterior subcapsular cataracts. Patients in both groups were matched for age and gender with no statistical difference (Table 1).

**Figure 1:**
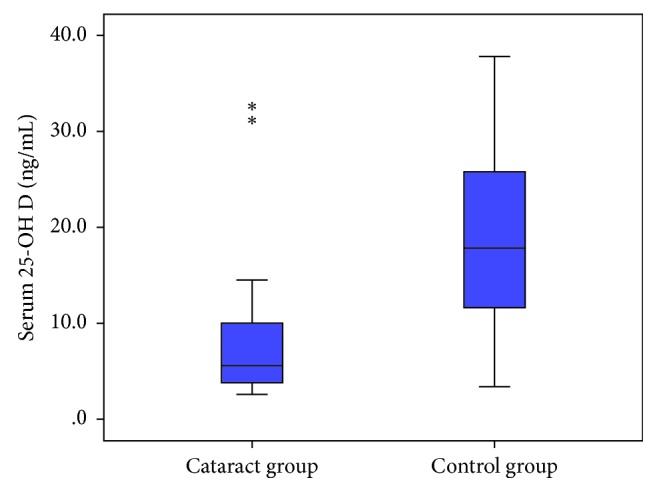
Box plot comparison of the serum 25-OH D (ng/mL) level in cases and control groups (*N*=710). ^*∗*^Outliers.

Mean 25-OH D level in cataract patients was outside the normal range as mean was 7.6 ng/mL ± 5.5 SD and median was 5.6 ng/mL (2.6–31.9) compared to age/sex-matched national standards for vitamin D levels. This serum 25-OH D level can be considered the severe deficiency category. However, the mean 25-OH D level in the control group was 18.5 ± 9.6 ng/mL, which is considered deficient as well (Figure 1).

High statistical significant difference was observed between both groups on comparing between genders and different age groups. The difference between serum 25-OH D among different cataract types was statistically significant, as shown in Table 2 (*P* value < 0.001). Patients with nuclear cataract showed the lowest level of 25-OH D, and the highest level was found in the posterior subcapsular type.

**Figure 2:**
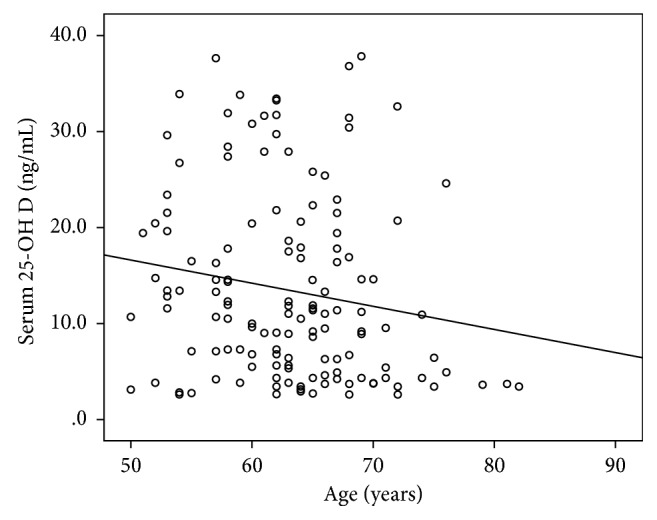
Correlation between the age and serum 25-OH D (ng/mL) among the study participants.

The correlation between the age and serum 25-OH D showed negative correlation which is statistically significant as the serum 25-OH D level is inversely related to increasing age, as shown in Figure 2.

## 5. Discussion

25-OH D deficiency showed inverse relationships with age-related macular degeneration (AMD) [16], diabetic retinopathy (DR) [17], uveitis, and dry eye disease [18]. Yet, 25-OH D serum levels were not investigated in relation to age-related cataracts in our locality nor on a large sample as well. Our study enrolled patients above 50 years of age with no history of chronic disease such as diabetes or hypertension that was found to have an inverse relationship with 25-OH D [19]. Our results represented far lower levels of serum 25-OH D in both control and cataract groups. The control group level of 25-OH D was lower than 30 ng/ml (18.5 ± 9.6 ng/mL), which indicates vitamin D deficiency according to the reference range and also lower than the reported levels in other studies which were conducted in different localities such as USA and Asia [18]. In addition, the cataract group was affected to a further extent and much lower than stated levels of severe deficiency (7.6 ± 5.5 ng/mL). These lower values may be attributed to the fact that the population of Upper Egypt are dark skinned who need more sunlight exposure to achieve average 25-OH D [20]. Another possible cause of discrepancy might be the different lifestyle with different dietary supplements. In a Korean study by Jee and Kim [21], the mean vitamin D concentration in cataract patients was 20.0 ng/mL in men and 17.5 ng/mL in women. This would lead us to realize that vitamin D differs with different localities and ethnic populations and also can be affected by latitude, sun exposure, and protection such as sun blocks or traditional clothing [22]. Vitamin D levels did not increase with age in either group. However, studies conducted in Korea and Thailand showed that elderly people had higher vitamin D levels compared with young people [23, 24], which was explained by more indoor office activities of the younger generation. Our results showed males having higher levels of vitamin D than females which might be explained by more sunlight exposure due to the nature of their outdoor activities.

The remarkable part of our results is that the lowest levels of serum 25-OH D were found in nuclear cataract patients which may give a novel information about relation of type of cataract to serum 25-OH D. This result correlates with Parks and Choi's [25] who found an inverse relationship between 25-OH D and nuclear cataract. Rao et al. and colleagues [26] reported the same result regarding the serum 25-OH D level in nuclear cataract in women with a certain age group of 50–69 years. Also Brown and Akaichi [15] found decreased serum 25-OH D in posterior subcapsular cataract. But further research is warranted to further investigate this finding as there are contradictory results from the previous studies [27, 28].

Our strict exclusion criteria of all compounding factors (such as chronic diseases and drug intake) that might affect the outcome added strength to our study. Yet, surveying larger number of cataract patients in future studies would be more conclusive. We also think that examining ocular 25-OH D would be useful to correlate with serum levels. Given the fact that vitamin D reduces inflammatory mediators and shows antioxidative functions [29–31], it would prove to be effective to administer it to protect against chronic diseases and cataract. Other studies regarding the effect of dietary vitamin D supplementation on prevention of cataract occurrence are needed; it was suggested previously that increased vitamin D intake may prevent cataract [21].

## 6. Conclusion

25-OH D levels in all enrolled individuals were below the reference levels with a severe deficiency in cataract patients. Nuclear cataract patients showed the lowest 25-OH D levels. These results might highlight the role of deficiency of 25-OH D in age-related cataract patients.

## Figures and Tables

**Table 1:** Sociodemographic characteristics in different study groups (*N*=710).

Parameter	Cataract group (*N*=325)	Control group (*N*=385)	*P* value
Age (years)			
Mean ± SD	63.1 ± 6.3	62.9 ± 6.7	0.93^*∗*^
Median (range)	63 (50–80)	63 (51–81)	

Age group (years)			
50–60	105 (32.3%)	135 (35.1%)	0.618^*∗∗*^
61–70	185 (56.9%)	205 (53.2%)	
> 71	35 (10.8%)	45 (11.7%)	

Sex			
Male (%)	155 (47.7%)	180 (46.8%)	0.821^*∗∗*^
Female (%)	170 (52.3%)	205 (53.2%)	

^*∗*^ *P* value was calculated by using the Mann–Whitney test; ^*∗∗*^*P* value was calculated using the chi-squared test.

**Table 2:** Comparison of the serum 25-OH D (ng/mL) level in the cataract group according to the type of cataract (*N*=325).

Parameter	Cortical (*N*=105) 32.3%	Nuclear (*N*=125) 38.5%	Posterior subcapsular (*N*=95) 29.2%	*P* value
Serum 25-OH D (ng/mL)				
Mean ± SD	7.2 ± 6.2	6.9 ± 6.1	9.8 ± 6.2	<0.001^*∗*^
Median (range)	5.3 (2.6–31.4)	4.3 (2.6–31.9)	9 (3.4–31.9)	

Comparison among the three groups has been done by the Kruskal–Wallis test. ^*∗*^Statistically significant.

## Data Availability

The data used to support the findings of this study are available from the corresponding author upon request.
